# Zinc complex of 3,5-di-tert-butyl salicylate inhibits viability, migration, and invasion in triple-negative breast cancer cells

**DOI:** 10.1038/s41598-022-08704-0

**Published:** 2022-03-16

**Authors:** Heng Chen, Dong Wang, Limei Fan, Zixin Liu, Weiran Zhang, Jinhua Xu, Yunyi Liu

**Affiliations:** School of Medicine, Jianghan University, Wuhan, 430056 Hubei China

**Keywords:** Cancer, Cell biology, Drug discovery

## Abstract

The zinc complex of 3,5-di-tert-butyl salicylate (Zn{[CH_3_)_3_C]_2_Sal}_2_^2−^) is a zinc ion chelate of salicylate. In this study, we found that this compound inhibits viability, invasion, and migration and induces apoptosis in triple-negative breast cancer 4T1 cells. RNA-seq showed that the expression of 17 genes was upregulated and 26 genes were downregulated significantly by Zn{[CH_3_)_3_C]_2_Sal}_2_^2−^ treatment. Further GO and KEGG analysis showed that the activity of Zn{[CH_3_)_3_C]_2_Sal}_2_^2−^ against triple-negative breast cancer cells may be involved in the JAK-STAT3, HIF-1, and TNF signaling pathways. The expression of key genes was verified by RT–PCR. The phosphorylation of STAT3 and its upstream SRC decreased drastically upon Zn{[CH_3_)_3_C]_2_Sal}_2_^2−^ treatment, as demonstrated by western blot. Our results indicate that Zn{[CH_3_)_3_C]_2_Sal}_2_^2−^ inhibits the activity of TNBC cells by downregulating the STAT3 signaling through the SRC pathway.

## Introduction

Breast cancer is the most common cancer among women and accounts for the second largest number of cancer deaths in women worldwide. Approximately 10 to 20% of new cases of breast cancer are triple-negative breast cancer (TNBC)^1,2^. TNBC does not express estrogen receptor (ER), progesterone receptor (PR) or human epidermal growth factor receptor 2 (HER2) and thus lacks therapeutic targets for endocrine therapy and HER2-directed agents^3^. Compared to other subtypes of breast cancer, TNBC is associated with a poorer prognosis due to a higher risk of metastasis and recurrence^4^. Apart from surgery and radiation therapy, cytotoxic chemotherapy remains the only FDA-approved method for treatment and the predominant form of systemic therapy for TNBC^5^. TNBC is sensitive to chemotherapy in the initial treatment^6^, but it eventually develops treatment resistance. Therefore, it remains crucial to find new chemotherapeutic agents for the treatment of TNBC.

Salicylic acids and their derivatives are mostly used as antipyretics, analgesics, and anti-inflammatory agents (categorized as nonsteroidal anti-inflammatory drugs) in clinical practice. They may also show certain therapeutic effects on tumors such as breast cancer^7^, pancreatic cancer^8^, lung cancer^9^, ovarian cancer and prostate cancer^10^. Small doses of aspirin can reduce the risk of multiple cancers, and aspirin inhibits the growth of various cancer cells in vitro*,* including breast cancer cells, through COX-dependent and COX-independent pathways^11^. This inhibition may be associated with the salicylic acid group. During the process of breast cancer metastasis, aspirin can resist platelet activation and thus inhibit platelet-induced epithelial transformation, migration, and invasion by breast cancer cells^12^. Aspirin can also inhibit the activation of transcription factor-1 (AP-1) and nuclear factor κB (NF-κB), thus affecting cellular viability, differentiation and apoptosis, transformation, invasion and metastasis of tumor cells^13^. However, there are few studies on the role of salicylate heavy metal chelates against cancer. Sorenson et al. found that copper salicylate and its derivatives can inhibit the growth and lung metastasis of ascites cancer in animal experiments, induce the differentiation of tumor cells, and prolong survival in animal models^14^. In addition, the combined use of copper salicylate can significantly improve the anticancer effect of cisplatin and reduce its toxicity. O'Connor et al. reported that the copper complex of salicylate inhibits the viability of MCF-7, DU145, HT29 and SKOV3 cancer cells^15^. Our previous study showed that the copper complex of phenanthroline salicylate can induce apoptosis of TNBC cells by downregulating the expression of antiapoptotic proteins such as Bcl-2, Bcl-xL and Survivin in vitro and *in vivo*^16^. Until now, the function of Zn{[CH_3_)_3_C]_2_Sal}_2_^2−^ has not been determined.

The activation of the signal transduction and transcription (STAT) protein family is closely related to the development of many tumors. Among the various STAT members, STAT3 is often overexpressed in tumor cells and tissue samples, and it regulates the expression of many oncogenes that control growth and metastasis^17^. As the intersection of several tumorigenic signaling pathways, STAT3 is considered to have an important role in tumor cells and the tumor microenvironment. The activation of STAT3 pathways is related to the aggressiveness of breast cancer and is involved in cell viability, apoptosis, metastasis and chemotherapy resistance. Both preclinical and clinical studies have shown that STAT3 plays a crucial role in the progression and metastasis of TNBC^18^.

In this study, we investigated the biological effect of Zn{[CH_3_)_3_C]_2_Sal}_2_^2−^ on TNBC. We found that a low concentration of Zn{[CH_3_)_3_C]_2_Sal}_2_^2−^ could inhibit the migration and invasion of TNBC cells. The STAT3 pathway was downregulated by Zn{[CH_3_)_3_C]_2_Sal}_2_^2−^ treatment, as analyzed by RNA-seq and western blot. This suggests that Zn{[CH_3_)_3_C]_2_Sal}_2_^2−^ may modulate the metastatic potential of TNBC by inhibiting the STAT3 signaling pathway.

## Results

### Effect of Zn{[CH_3_)_3_C]_2_Sal}_2_^2−^ on the viability of TNBC cells

**Figure 1:**
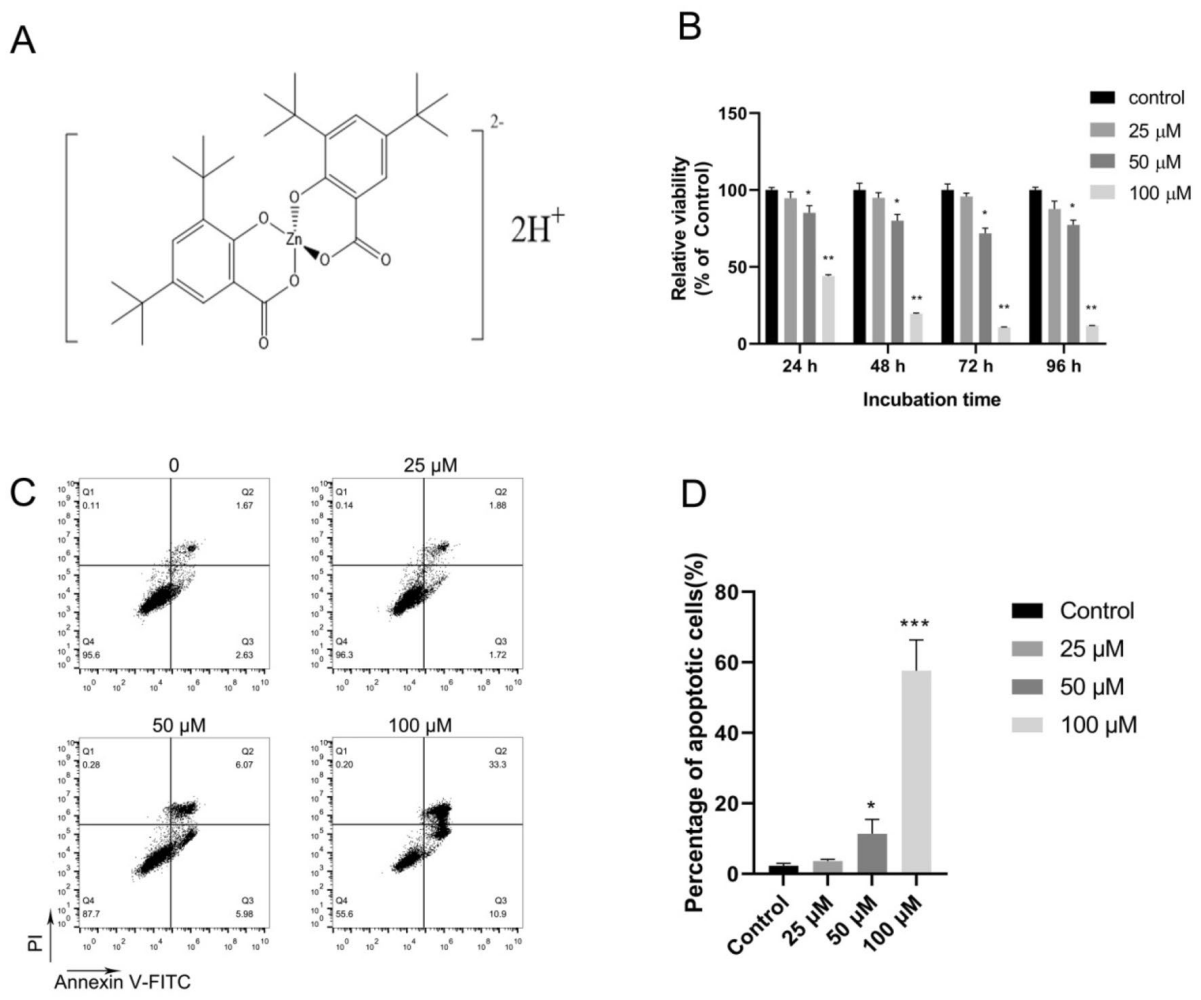
Zn{[CH_3_)_3_C]_2_Sal}_2_^2−^ inhibits cell viability and induces apoptosis of TNBC 4T1 cells. (**A**) Structure of the zinc complex of 3,5-di-tert-butyl salicylate. (**B**) Cell viability was decreased by Zn{[CH_3_)_3_C]_2_Sal}_2_^2−^ treatment. 4T1 cell viability was determined by MTS assay after Zn{[CH_3_)_3_C]_2_Sal}_2_^2−^ treatment. The statistical analysis was conducted by comparing the OD_492_ value of each treatment concentration with that of the DMSO control. Error bars represent mean ± SEM. *P < 0.05, **P < 0.01. (**C**) Zn{[CH_3_)_3_C]_2_Sal}_2_^2−^ induced apoptosis of 4T1 cells in a concentration-dependent manner. 4T1 cells were incubated with the indicated concentrations of Zn{[CH_3_)_3_C]_2_Sal}_2_^2−^ for 24 h, and apoptosis was measured by flow cytometry after staining with Annexin V-PI. (**D**) Statistical analysis of apoptosis in three independent experiments. Error bars represent mean ± SEM. *P < 0.05, ***P < 0.001.

Zn{[CH_3_)_3_C]_2_Sal}_2_^2−^ is a zinc ion chelate of salicylate, and its molecular structure is shown in Fig. 1A. To examine the effect of Zn{[CH_3_)_3_C]_2_Sal}_2_^2−^ on TNBC cell viability, TNBC 4T1 cells were incubated with various concentrations of Zn{[CH_3_)_3_C]_2_Sal}_2_^2−^, and then cell viability was measured by MTS assay. As shown in Fig. 1B, 4T1 cell viability was not affected by 25 µM Zn{[CH_3_)_3_C]_2_Sal}_2_^2−^ treatment. However, the viability decreased dramatically with higher doses of Zn{[CH_3_)_3_C]_2_Sal}_2_^2−^ treatment. After Zn{[CH_3_)_3_C]_2_Sal}_2_^2−^ treatment, the IC_50_ of 4T1 cells was 88.459 µM (83.938–92.98),68.752 µM (64.421–73.082) and 61.784 µM (60.14–63.427) at 24 h, 48 h and 72 h, respectively. We analyzed the effect of Zn{[CH_3_)_3_C]_2_Sal}_2_^2−^ on the viability of the human TNBC cell line MDA-MB-231 and observed similar results to 4T1 (Fig. S1). These results suggest that Zn{[CH_3_)_3_C]_2_Sal}_2_^2−^ inhibits TNBC cell viability in a time- and concentration-dependent manner.

Since the viability of 4T1 cells was decreased by Zn{[CH_3_)_3_C]_2_Sal}_2_^2−^, we then analyzed the effects of Zn{[CH_3_)_3_C]_2_Sal}_2_^2−^ on the induction of apoptosis of 4T1 cells. 4T1 cells were treated with different concentrations of Zn{[CH_3_)_3_C]_2_Sal}_2_^2−^ for 24 h, and the degree of apoptosis was analyzed by Annexin V-PI staining and flow cytometry. As shown in Fig. 1C and D, less than 5% apoptosis was detected with a 25 µM Zn{[CH_3_)_3_C]_2_Sal}_2_^2−^ concentration. Treatment with 50 and 100 µM Zn{[CH_3_)_3_C]_2_Sal}_2_^2^ significantly induced apoptosis. This indicates that decreased viability by Zn{[CH_3_)_3_C]_2_Sal}_2_^2−^ treatment is due to apoptosis induction. This experiment suggests that a lower concentration of (< 25 µM) Zn{[CH_3_)_3_C]_2_Sal}_2_^2−^ has little effect on cell viability.

### Zn{[CH_3_)_3_C]_2_Sal}_2_^2−^ inhibits the migration and invasion of 4T1 cells

**Figure 2:**
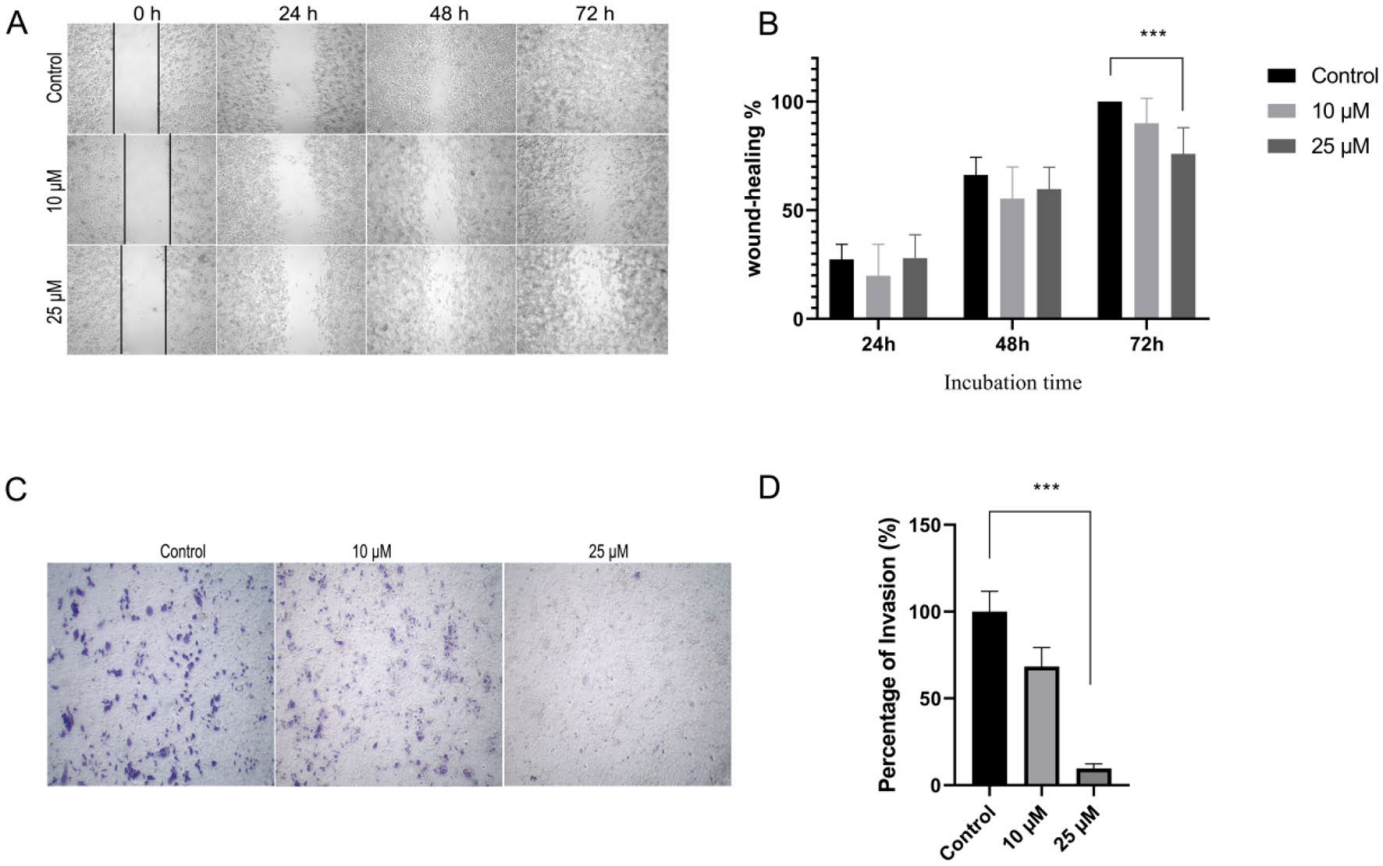
Zn{[CH_3_)_3_C]_2_Sal}_2_^2−^ inhibits 4T1 cell migration and invasion. (**A**) With 10 and 25 μM Zn{[CH_3_)_3_C]_2_Sal}_2_^2−^ treatment, 4T1 cell migration ability was determined by wound healing assay at 0 h, 24 h, 48 h, and 72 h. (**B**) Statistical analysis of the wound healing assay of three independent experiments. Error bars represent the mean ± SEM, ***P < 0.001. C) The invasion ability was determined by a Transwell invasion assay after 22 h incubation. (**D**) Statistical analysis was performed with three independent Transwell invasion assays. Error bars represent mean ± SEM. ***P < 0.001.

Since migration is critical for metastasis, we investigated the inhibitory effect of Zn{[CH_3_)_3_C]_2_Sal}_2_^2−^ on 4T1 cell migration through a wound healing assay. As shown in Fig. 2A and B wound healing was hindered by 25 µM Zn{[CH_3_)_3_C]_2_Sal}_2_^2−^ treatment compared with the control, and the effect was statistically significant. We then investigated whether Zn{[CH_3_)_3_C]_2_Sal}_2_^2−^ could influence the invasive capacity of 4T1 cells, which is a key step of metastasis. Transwell invasion assays were carried out after 24 h Zn{[CH_3_)_3_C]_2_Sal}_2_^2−^ treatment. As shown in Fig. 2C and D, invasion was reduced by 25 µM Zn{[CH_3_)_3_C]_2_Sal}_2_^2−^ treatment compared with the control, and the effect was statistically significant. All of these results indicate that Zn{[CH_3_)_3_C]_2_Sal}_2_^2−^ inhibits the migration and invasion of 4T1 cells in a dose-dependent manner.

### Gene expression changes in 4T1 cells after Zn{[CH_3_)_3_C]_2_Sal}_2_^2−^ treatment

**Figure 3:**
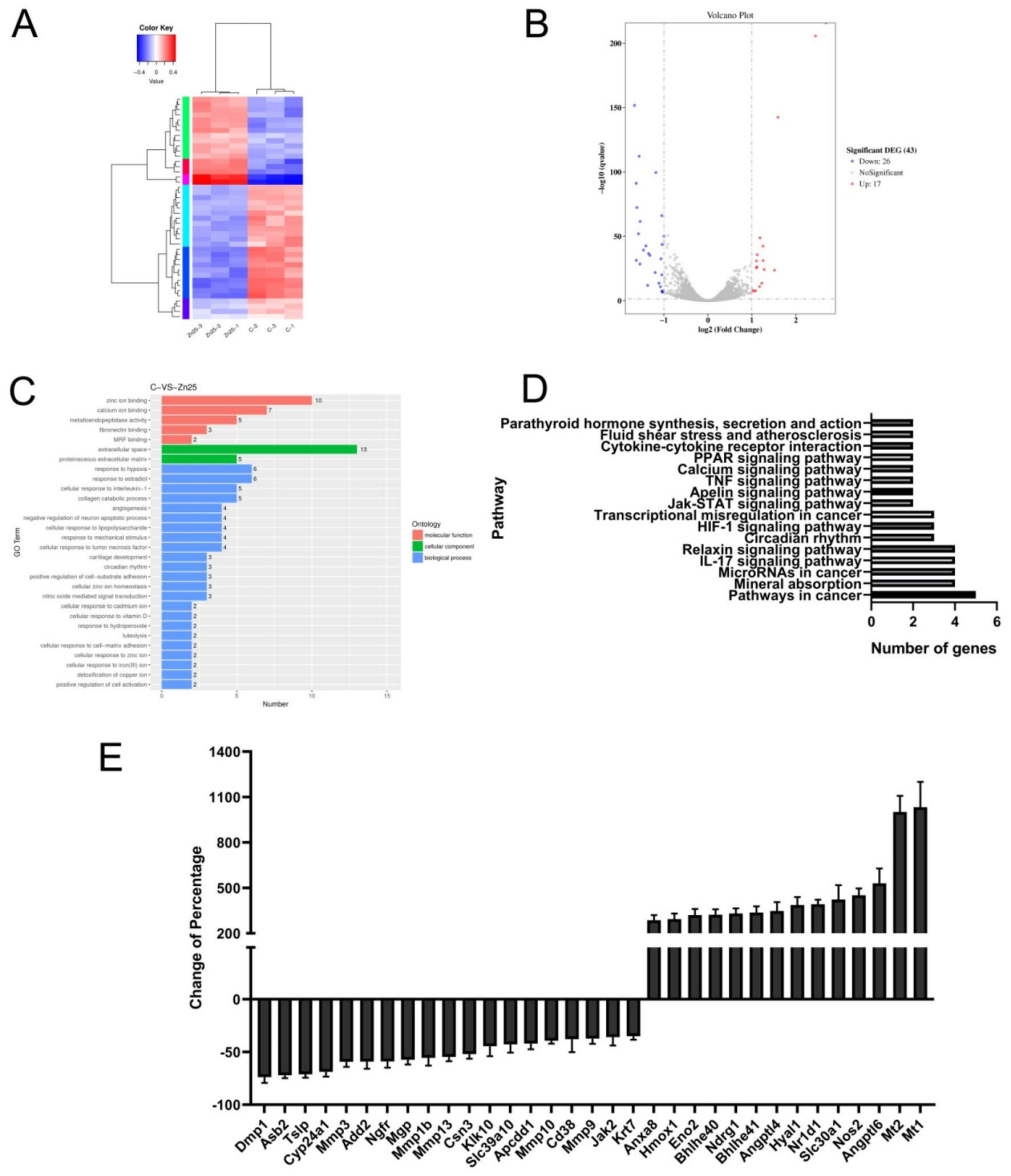
Gene expression changes in 4T1 cells after Zn{[CH_3_)_3_C]_2_Sal}_2_^2−^ treatment analyzed by RNA-seq. (**A**) Hierarchical clustering of differentially expressed mRNAs between control and Zn{[CH_3_)_3_C]_2_Sal}_2_^2−^ (25 μM)-treated 4T1 cells. (**B**) A volcano plot shows differentially expressed RNAs after treating 4T1 cells with Zn{[CH_3_)_3_C]_2_Sal}_2_^2−^. (**C**) The enriched cell functional classifications for the DEGs in 4T1 cells after treatment with Zn{[CH_3_)_3_C]_2_Sal}_2_^2−^. (**D**) The top 16 enriched pathways for the DEGs in 4T1 cells after treatment with Zn{[CH_3_)_3_C]_2_Sal}_2_^2−^. (**E**) mRNA expression levels in Zn{[CH_3_)_3_C]_2_Sal}_2_^2–^treated 4T1 cells verified by RT–PCR.

To further examine the molecular mechanism of Zn{[CH_3_)_3_C]_2_Sal}_2_^2−^ against 4T1 cells, RNA-seq analysis was conducted. After Zn{[CH_3_)_3_C]_2_Sal}_2_^2−^ treatment, the expression level of 4965 genes was changed significantly (*p* < 0.05). Among the abovementioned differentially expressed genes, 43 genes exhibited greater than twofold changes, of which 17 genes were upregulated and 26 genes were downregulated (Fig. 3A,B).

### Enrichment analysis of differentially expressed genes

The significantly differentially expressed genes (SDEGs) between the control and Zn{[CH_3_)_3_C]_2_Sal}_2_^2−^ treated 4T1 cells were subjected to functional GO and KEGG pathway analyses. For GO classification, three different categories, molecular function (MF), cellular component (CC), and biological process (BP), were used to enrich the SDEGs. As shown in Fig. 3C, among the MF category terms, the SDEGs were primarily enriched in zinc ion binding, calcium ion binding, metalloendopeptidase activity, fibronectin binding, and MRF binding. Among the CC terms, they were predominantly enriched in the anchored component of extracellular space and proteinaceous extracellular matrix. Among the BP terms, the SDEGs were mainly enriched in terms of response to hypoxia and estradiol, cellular response to interleukin-1, collagen catabolic process and angiogenesis. The top 10 of the 23 KEGG pathways identified as enriched are presented in Fig. 3D. Pathways in cancer ranked the highest. Numerous well-established signaling pathways were also enriched, including the JAK-STAT signaling pathway, HIF-1 signaling pathway, and TNF signaling pathway.

### Verification of gene expression change by RT–PCR

To confirm the RNA-seq results, the expression levels of some genes of interest were verified by RT–PCR. As expected, the RT–PCR results were consistent with the RNA-seq analysis. As shown in Fig. 3E, the mRNA expression levels of MT1 and MT2, which are related to metal ion transportation, were upregulated nearly tenfold. The expression of Angpt16 and Angp14, which are closely related to angiogenesis, was also significantly upregulated. Meanwhile, Mmp3, Mmp9, Mmp16 and Mmp10, which belong to the MMP family and are closely related to tumor metastasis, were downregulated. In addition, JAK2, which is an important component of the STAT signaling pathway, was downregulated significantly.

### Effects of Zn{[CH_3_)_3_C]_2_Sal}_2_^2−^ on the JAK-STAT signaling pathway, Src pathway and PI3K pathway

**Figure 4:**
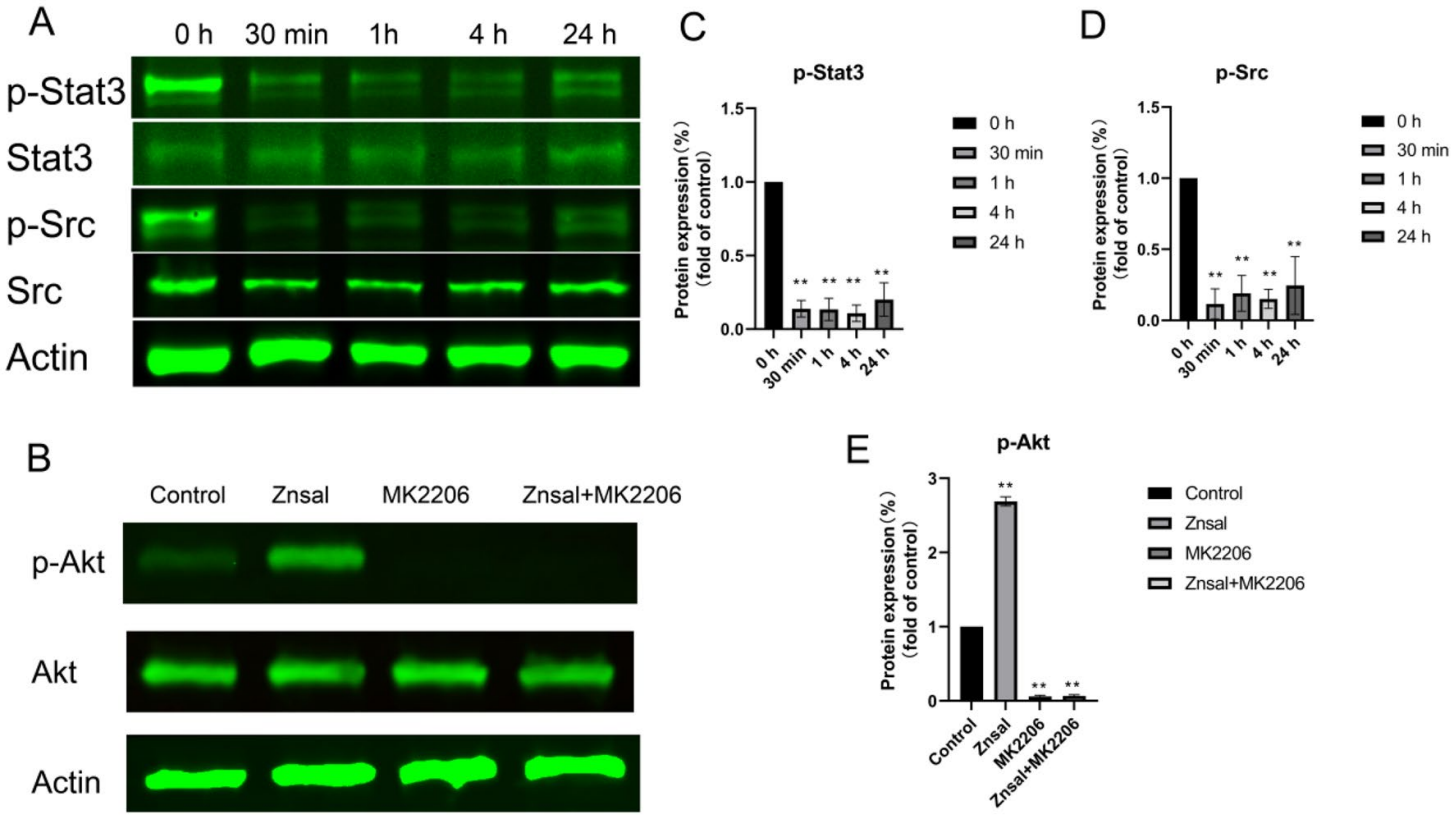
Zn{[CH_3_)_3_C]_2_Sal}_2_^2−^ inhibits the activation of the STAT3 signaling pathway and SRC pathway. (**A**) The protein expression levels of STAT3, p-STAT3, p-Src, and Src at different time points in 4T1 cells treated with Zn{[CH_3_)_3_C]_2_Sal}_2_^2−^ were analyzed by western blot. (**B**) The protein expression levels of Akt and p-Akt were analyzed by western blot after 30 min of treatment with Zn{[CH_3_)_3_C]_2_Sal}_2_^2−^ or MK2206 alone or in combination. (**C**–**E**) Expression changes (fold change relative to control) of p-STAT3, p-Src and p-Akt^473^ were analyzed by densitometry with the results of two independent Western blots. Data are presented as the mean ± SEM (*P < 0.05, **P < 0.01).

The JAK-STAT3 signaling pathway is a multifunctional pathway in cancer progression that is involved in cancer cell viability, migration, invasion, and even apoptosis. Based on the RNA-seq and RT–PCR results, we believe that the JAK-STAT signaling pathway is involved in the anticancer effect of Zn{[CH_3_)_3_C]_2_Sal}_2_^2−^. To test our hypothesis, protein expression and phosphorylation were analyzed in 4T1 cells after Zn{[CH_3_)_3_C]_2_Sal}_2_^2−^ treatment. As shown in Fig. 4A, the expression levels of Stat3 and Src protein were not affected by treatment with 25 μM Zn{[CH_3_)_3_C]_2_Sal}_2_^2−^. However, the phosphorylation of these two proteins was reduced dramatically after 30 min of Zn{[CH_3_)_3_C]_2_Sal}_2_^2−^ treatment. It was still far below the control level after 24 h of treatment (Fig. 4A,C,D). We also observed that p-Stat3 was downregulated by Zn{[CH_3_)_3_C]_2_Sal}_2_^2−^ treatment in MDA-MB-231 cells (Fig. S2). Unexpectedly, we found that phosphorylation of JAK2 was not affected by Zn{[CH_3_)_3_C]_2_Sal}_2_^2−^ treatment in 4T1 cells (Fig. S3). These results demonstrated that Zn{[CH_3_)_3_C]_2_Sal}_2_^2−^ inhibits the activation of STAT3 through the Src pathway. In addition, we found that pAKT^473^ was significantly increased after 30 min of treatment with 25 μM Zn{[CH_3_)_3_C]_2_Sal}_2_^2−^ (Fig. 4B). To further explore the relationship of Zn{[CH_3_)_3_C]_2_Sal}_2_^2−^ to AKT, 4T1 cells were treated with 25 μM Zn{[CH_3_)_3_C]_2_Sal}_2_^2−^ together with an AKT inhibitor (MK2206). As shown in Fig. 4B and E, combination treatment completely suppressed pAKT^473^.

### Effect of Zn{[CH_3_)_3_C]_2_Sal}_2_^2−^ on lung metastasis in vivo

**Figure 5:**
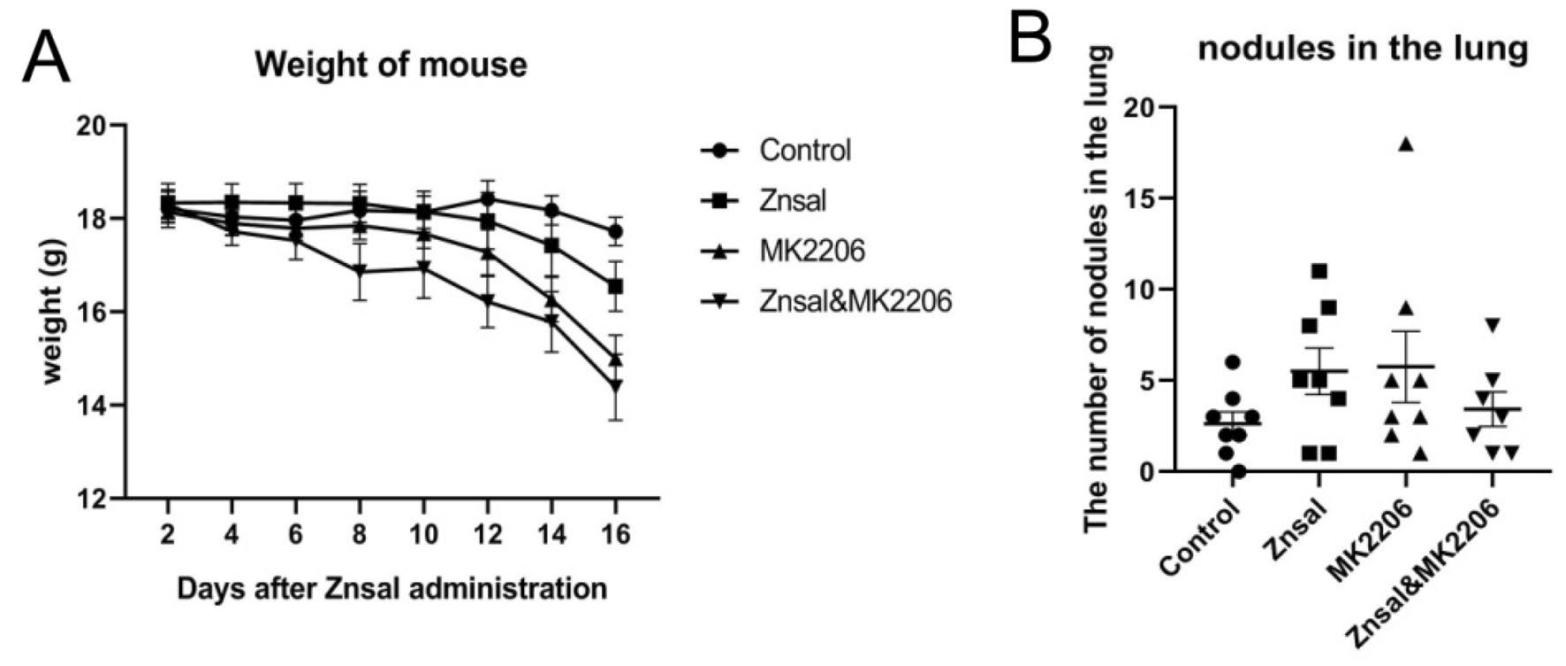
Effect of Zn{[CH_3_)_3_C]_2_Sal}_2_^2−^ on lung metastasis in vivo*.* Lung tumorigenesis was established by injecting 4T1 cells through the tail vein of BALB/c mice. (**A**) Changes in mouse body weight after 16 days of treatment with Zn{[CH_3_)_3_C]_2_Sal}_2_^2−^ or MK2206 alone or in combination. (**B**) Number of tumor nodules in the mouse lung after 16 days of Zn{[CH_3_)_3_C]_2_Sal}_2_^2−^ or MK2206 treatment alone or in combination.

Lung metastasis was measured at Day 16. As shown in Fig. 5B, the pulmonary metastases in the zinc salicylate treatment group and MK2206 treatment group were similar to those in the control group. In addition, no inhibitory effect on lung metastasis was observed in the group treated with Zn{[CH_3_)_3_C]_2_Sal}_2_^2−^ and MK2206. The weight of Zn{[CH_3_)_3_C]_2_Sal}_2_^2−^ treated mice decreased slightly compared with that of the control group. Treatment with the Akt inhibitor MK2206 alone or in combination with Zn{[CH_3_)_3_C]_2_Sal}_2_^2^ caused significant weight loss (Fig. 5A).

## Discussion

The prognosis of TNBC is poor because of its high rate of relapse and metastasis. Drugs that target metastasis are of great significance. In this study, we demonstrated that low-dose Zn{[CH_3_)_3_C]_2_Sal}_2_^2−^ inhibits the migration and invasion of 4T1 mouse TNBC cells, while high-dose Zn{[CH_3_)_3_C]_2_Sal}_2_^2−^ inhibits cell viability and induces apoptosis. In addition, we explored the anti-TNBC mechanism of Zn{[CH_3_)_3_C]_2_Sal}_2_^2−^ using RNA-seq and bioinformatic analyses. Furthermore, we demonstrated that Zn{[CH_3_)_3_C]_2_Sal}_2_^2−^ can inhibit activation of the STAT3 signaling pathway by western blot analysis.

By utilizing GO and KEGG pathway analyses, we found changes in gene expression associated with zinc ion binding, metalloendopeptidase activity, fibronectin binding, proteinaceous extracellular matrix, collagen catabolic process, and angiogenesis in 4T1 cells. The RT–PCR results verified that the transcription levels of genes related to metal ion transport, angiogenesis, and tumor metastasis were significantly altered. This indicates that the antitumor effect of Zn{[CH_3_)_3_C]_2_Sal}_2_^2−^ may be related to those genes. The pathway analyses indicated that many enriched pathways were associated with viability, migration, and invasion, which was consistent with our functional analyses. For instance, the JAK-STAT signaling pathway is important in tumor metastasis. The establishment of a database of RNA expression after Zn{[CH_3_)_3_C]_2_Sal}_2_^2−^ treatment in 4T1 cell lines could provide a broad foundation to guide focused studies of TNBC research.

The mechanism of metastasis in TNBC is regulated by multiple signaling pathways: the NF-κB pathway^19^, Wnt/beta-Catenin pathway^20^, PI3K/AKT pathway, FAK/c-Src pathway^21^, and JAK/STAT3 pathway. The STAT pathway is the focal point of multiple signal transduction cascades. STAT3 activation is short and strictly controlled, but STAT3 protein appears constitutively activated in multiple tumor cells^22^. Specifically, studies have shown that STAT3 is frequently activated in TNBC and is an integral part of tumorigenesis and metastasis^23^. There are multiple mechanisms involved in STAT3 regulation. Upon cytokine or growth factor binding to the membrane receptor, JAK proteins are activated and phosphorylate STAT3 at Tyr705. Activated STAT3 then translocates to the nucleus and upregulates the transcription of downstream genes. In addition, nonreceptor tyrosine kinases such as Src can phosphorylate and activate STAT3^24^. By analyzing the transcriptional differences between TNBC cells and normal breast cells, Zhan et al. and other researchers observed abnormal expression of multiple signaling proteins in the IL6/JAK2/STAT3 pathway in TNBC, and constitutive activation of JAK2/STAT3 may be an important factor in metastasis of TNBC^25^. The tyrosine kinase SRC also plays an important role in cell viability^26^, the cell cycle, cell adhesion^27^, migration, and invasion signaling pathways. SRC is overexpressed or highly active in various solid tumor cell lines and tissues, and SRC inhibitors abrogate breast cancer cell invasion and metastasis^28^. STAT3 is activated by both SRC and JAK2 in breast cancer^22^. In our study, we found that Zn{[CH_3_)_3_C]_2_Sal}_2_^2−^ treatment caused downregulation of phosphorylation of SRC and STAT3 but not JAK2. It indicates that STAT3 is inhibited through the SRC pathway, which consequently affects the migration and invasive ability of 4T1 TNBC cells. The PI3K pathway promotes tumorigenesis independent of JAK-STAT3^29^. Indeed, we found that Zn{[CH_3_)_3_C]_2_Sal}_2_^2−^ treatment caused upregulation of p-Akt^473^, and combination treatment with Zn{[CH_3_)_3_C]_2_Sal}_2_^2−^ and an AKT inhibitor completely suppressed pAKT^473^. This suggests that a combination of STAT3 inhibitor and an inhibitor of the PI3K signaling pathway will be an effective strategy against tumors with both PI3K and STAT3 activation. To date, there are no FDA-approved STAT3 inhibitors for clinical use. Our results indicate that Zn{[CH_3_)_3_C]_2_Sal}_2_^2−^ warrants further study as a potential therapeutic drug for TNBC.

We did not observe an antimetastatic effect of Zn{[CH_3_)_3_C]_2_Sal}_2_^2−^ in an animal study, and we consider that the drug was not delivered to the tumor properly. Further study with nanoparticle delivery is warranted.

## Materials and methods

### Materials

Zn{[CH_3_)_3_C]_2_Sal}_2_^2−^ was synthesized by Dinglong Chemicals (Wuhan, Hubei, China). It was dissolved in dimethyl sulfoxide (DMSO) as a stock solution and further diluted with medium to specific concentrations. Lipofectamine 2000, Roswell Park Memorial Institute (RPMI) 1640 (RPMI-1640), fetal bovine serum (FBS), and penicillin–streptomycin solution were purchased from Gibco-BRL-Life Technologies (Grand Island, NY). Primers were ordered from Genewiz. Actin, Stat3, Src, phospho-Jak2, phospho-Src, phospho-Akt and Akt antibodies were obtained from Cell Signaling Technology (Danvers, MA, USA). Phospho-Stat3 antibody was purchased from Abcam (Cambridge, UK).

### MTS assay

To detect the effect of Zn{[CH_3_)_3_C]_2_Sal}_2_^2−^ on TNBC cell viability, 7,500 cells/well were plated in 96-well plates, incubated overnight to allow adhesion, and then treated with different concentrations of Zn{[CH_3_)_3_C]_2_Sal}_2_^2−^ for the indicated durations. Twenty microliters of 3-(4,5-dimethylthiazol-2-yl)-5-(3-carboxymethoxyphenyl)-2-(4-sulfophenyl)-2H-tetrazolium (MTS) was added to the culture medium for 3 h at 37 °C. The optical density was read at 492 nm on a microplate reader (Biotek, VT). Quintuplicate wells were measured in each treatment group. Cell viability was calculated as (OD_treated _− OD_blank_)/(OD_control _− OD_blank_). The IC_50_ was obtained with Graph Pad calculation.

### Cell apoptosis detection

Apoptosis of 4T1 cells was determined by flow cytometry with Annexin V-FITC/PI staining. Cells were plated into six-well plates. After treatment with Zn{[CH_3_)_3_C]_2_Sal}_2_^2−^ for 24 h, cells were washed with PBS, harvested and further stained according to the manufacturer’s instructions. Analysis was performed using flow cytometry (BD Biosciences, San Jose, CA, USA). The data was further analyzed by BD FlowJo.

### Wound healing assay

Logarithmic growth phase cells were plated into six-well plates and allowed to reach 90% confluence. A "scratch" was made by a 10 µl pipette tip, and then different concentrations of Zn{[CH_3_)_3_C]_2_Sal}_2_^2−^ were added. A photograph of the "scratch" fusion was taken in the Zn{[CH_3_)_3_C]_2_Sal}_2_^2−^ treated group and the control group every 12 h. ImageJ 1.8.0 software (https://imagej.en.softonic.com/download) was used for image data analysis.

### Cell invasion assay

Transwell chambers with Matrigel-coated membranes were placed in 24-well plates and incubated with 500 µl serum-free medium at 37 °C for 2 h. Then, 4T1 cells (1 × 10^5^) in 500 µl of serum-free medium were seeded into the top chamber, and 750 µl of RPMI-1640 medium with 10% FBS was added into the lower compartment of the Transwell chambers as the chemical attractant. After incubation for 22 h at 37 °C, the cells were fixed and stained with crystal violet. The invaded cells in five randomly chosen fields of each well were counted. The invasion index was calculated as treated/control.

### RNA-sequencing

cDNA library construction, library purification and transcriptome sequencing were carried out according to the instructions of Suzhou GENEWIZ Company. Three samples in each group were used for the RNA-sequencing assay.

### GO and KEGG pathway enrichment analysis

Differential expression analysis was performed using the DESeq2 Bioconductor package, a model based on the negative binomial distribution. The estimates of dispersion and logarithmic fold changes incorporated data-driven prior distributions, and the Padj of genes was set to < 0.05 to detect differentially expressed genes. GOSeq (v1.34.1) was used to identify Gene Ontology (GO) terms that annotate a list of enriched genes with a significant padj less than 0.05. In addition, top GO was used to plot DAG. KEGG (Kyoto Encyclopedia of Genes and Genomes) is a collection of databases dealing with genomes, biological pathways, diseases, drugs, and chemical substances (http://en.wikipedia.org/wiki/KEGG). We used scripts in house to enrich significantly differentially expressed genes in KEGG pathways.

### RNA extraction and quantitative real-time RT–PCR

Total RNA was extracted from 4T1 cells using TRIzol reagent (Invitrogen, Carlsbad, CA, USA) to analyze differentially expressed genes. cDNA was prepared from 1 μg of total RNA using a SuperScript III First-Strand Synthesis System kit (Invitrogen) according to the manufacturer’s instructions. Quantitative real-time polymerase chain reaction (qRT–PCR) was carried out in a CFX Connect Real-time System (Bio–Rad, Singapore) using iTaq Universal SYBR Green Supermix (Hercules, CA, USA). The primers used in this study are listed in Supplemental Table 1. The 2^(−ΔΔCt)^ method was used to analyze relative expression changes of mRNA, and β-actin was used as an internal reference.

### Western blotting assay

After treatment with different concentrations of Zn{[CH_3_)_3_C]_2_Sal}_2_^2−^ or MK2206 (AKT inhibitor) for a specific period of time, the cell protein was extracted according to the experimental instructions, separated by SDS–PAGE, and transferred to a nitrocellulose filter membrane (Pall, Pensacola, USA). The membranes were incubated with the specific primary antibody and then incubated with IRDye secondary antibodies (Licor, NB, USA). The membranes were scanned, and images were captured with Odyssey SA (Licor, NB, USA). Image Studio Ver 5.2 was used to quantify the intensities of the immunoreactive bands.

### Animal model for lung metastasis

Animal experiments followed the rules of the animal ethics committee of Jianghan University and were performed in accordance with relevant guidelines and regulations, including ARRIVE guidelines. The experimental mice were purchased from Wuhan Wanqian Jiaxing Biotechnology Co., Ltd. To establish the tumor model, 4T1 cells (5 × 10^4^ cells in 100 μl volume) were injected through the tail vein of 6-week-old female BALB/c mice. Zn{[CH_3_)_3_C]_2_Sal}_2_^2−^ (15 mg/kg) was administered by intraperitoneal injection three times a week after transplantation. For AKT inhibitor, mice were gavaged with 0.1 mL MK2206 (120 mg/kg) three times a week. Combination treatment was done accordingly. Animals were humanely sacrificed on Day 16 after 4T1 transplantation, and the number of tumor nodules in the lungs was recorded for analysis of metastasis.

### Statistical analysis

All statistical analyses were performed using GraphPad Prism 9 software (https://www.graphpad.com/). Comparisons between two groups were performed using Student’s t test. Statistical significance was defined as *p* < 0.05. Data are expressed as the mean ± standard error of the mean; n ≥ 3, unless otherwise stated.

### Ethics approval

Animal experiments were approved by the animal ethics committee of Jianghan University.

### Statement of animal experiments

All experimental procedures conformed to the guidelines of Care and Use of Laboratory Animals of China for animal experimentation. All animal handling methods were carried out in accordance with relevant guidelines and regulations. And the study was carried out in compliance with the ARRIVE guidelines.

## Supplementary Information

Supplementary Information.
