# Supplementary material for: Zinc complex of 3,5-di-tert-butyl salicylate inhibits viability, migration, and invasion in triple-negative breast cancer cells
Source: Sci Rep. 2022 Mar 16;12:4545. doi: 10.1038/s41598-022-08704-0 (PMC8927491; doi:10.1038/s41598-022-08704-0)
Supplement: Supplementary file 1 — Supplementary Information. [file 41598_2022_8704_MOESM1_ESM.pdf]

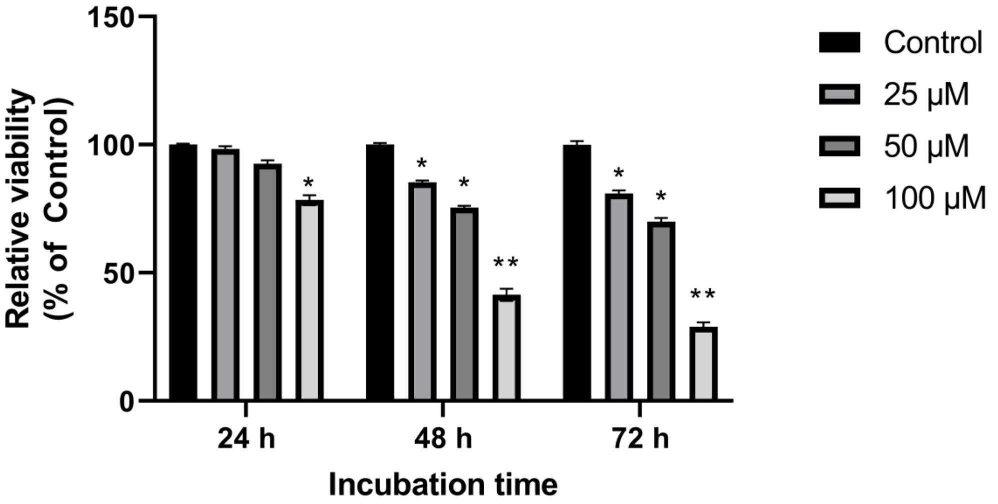

Fig. S1 MDA-MB-231 cell viability was decreased by $Zn\{[(CH_3)_3C]_2Sal\}_2^{2-}$ treatment. MDA-MB-231 cell viability was determined by MTS assay after $Zn\{[(CH_3)_3C]_2Sal\}_2^{2-}$ treatments. The statistical analysis was conducted by comparing the OD_{492} value of each treatment concentration with that of DMSO control. Error bars represent mean ± SEM. *P < 0.05, **P < 0.01.

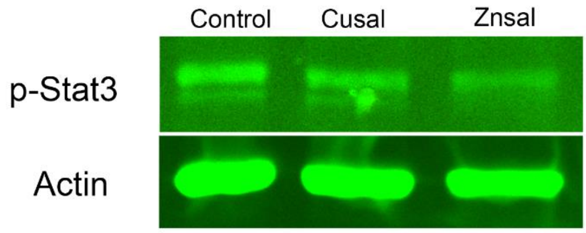

Fig. S2 The expression of p-Stat3 was downregulated by $Zn\{[(CH_3)_3C]_2Sal\}_2^{2-}$ treatment in MDA-MB-231 cells, as analyzed by western blotting. MDA-MB-231 cells were treated with $Zn\{[(CH_3)_3C]_2Sal\}_2^{2-}$ or DMSO (control) for 24hr. Actin was used as loading control.

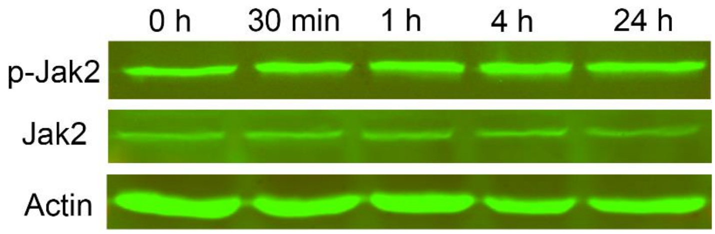

Fig. S3 $Zn\{[(CH_3)_3C]_2Sal\}_2^{2-}$ has no effect on the expression of p-Jak2 and total Jak2 in 4T1 cells. 4T1 cells were treated with $Zn\{[(CH_3)_3C]_2Sal\}_2^{2-}$ for Specific time and protein expression was analyzed by western blotting. Actin was used as loading control.

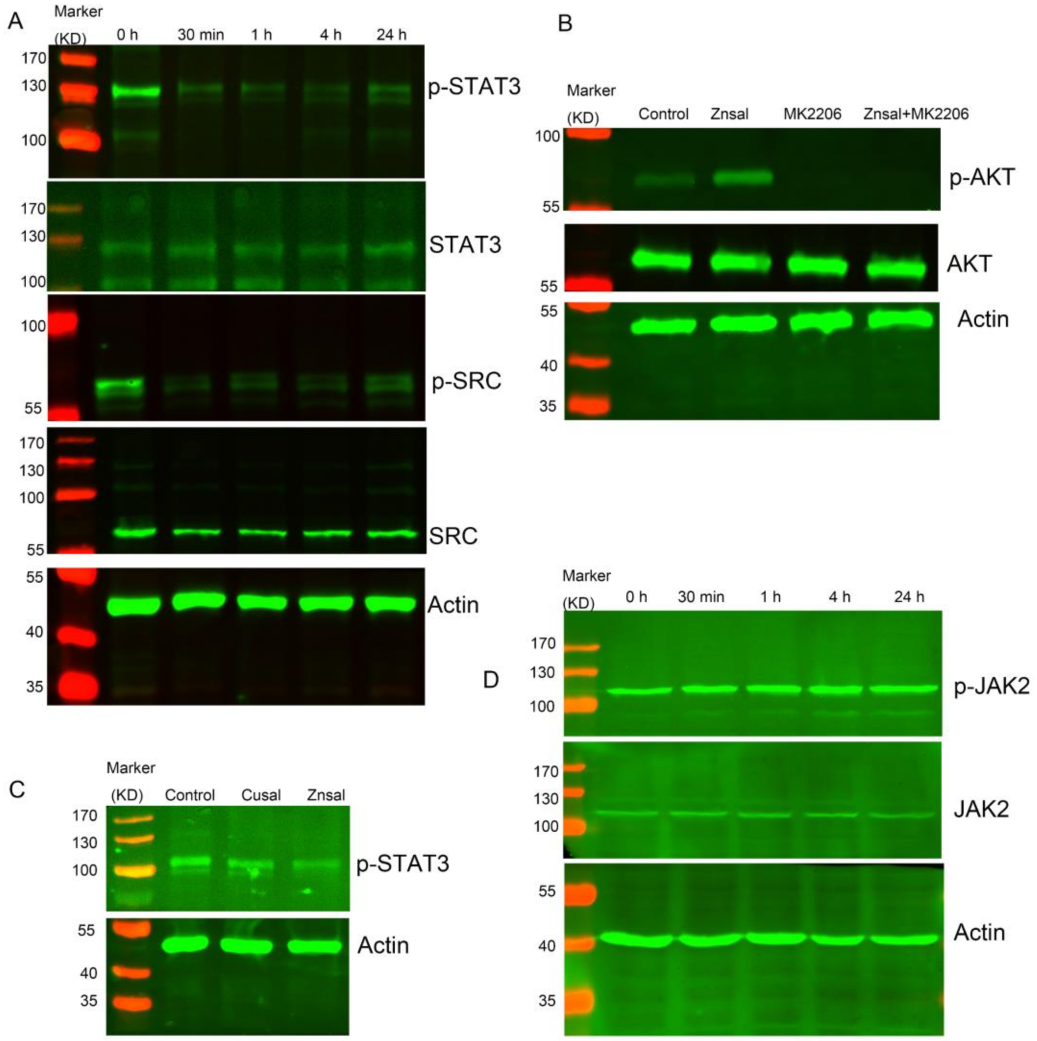

Fig. S4 A-B) The original images of Western blot in Fig.4.

C) The original images of Western blot in Fig.S2.

D) The original images of Western blot in Fig.S3.

qPCR	Forward (5′-3′)	Reverse (5′-3′)
Mt2	GCCTGCAAATGCAAACAATGC	AGCTGCACTTGTCGGAAGC
Mt1	AAGAGTGAGTTGGGACACCTT	CGAGACAATACAATGGCCTCC
Mmp13	TGTTTGCAGAGCACTACTTGAA	CAGTCACCTCTAAGCCAAAGAAA
Nos2	GGAGTGACGGCAAACATGACT	TCGATGCACAACTGGGTGAAC
Mmp3	GGCCTGGAACAGTCTTGGC	TGTCCATCGTTCATCATCGTCA
Mmp9	GGACCCGAAGCGGACATTG	CGTCGTCGAAATGGGCATCT
Mpg	AGCCCAAAAGAGAGTCCAGGA	TGCCTGAAGTAGCGGTTGTAG
Tslp	ACGGATGGGGCTAACTTACAA	AGTCCTCGATTTGCTCGAACT
JAK2	GGAATGGCCTGCCTTACAATG	TGGCTCTATCTGCTTCACAGAAT
Ngfr	TGCCGATGCTCCTATGGCTA	CTGGGCACTCTTCACACACTG
Krt7	AGGAGATCAACCGACGCAC	GTCTCGTGAAGGGTCTTGAGG
Ndrg1	ATGTCCCGAGAGCTACATGAC	CCTGCTCCTGAACATCGAACT
Apcdd1	GAAAGAGGTTGGGGTGAGACT	GAAGCCGAATCAAGCTGGTAA
Csn3	ATTCTGGCATTAACTCTGCCC	AAAGATGGCCTGTAGTGGTAGTA
Nr1d1	TACATTGGCTCTAGTGGCTCC	CAGTAGGTGATGGTGGGAAGTA
Add2	CCGACAAGACATAAAGTCAGCC	TGATACACTTCCCCTTCGGTT
Mmp10	GAGCCACTAGCCATCCTGG	CTGAGCAAGATCCATGCTTGG
Eno2	AGGTGGATCTCTATACTGCCAAA	GTCCCCATCCCTTAGTTCCAG
Slc39a10	TCATCGCCATCGTTTGCATCA	CTCTGGTGAAGGGCTGTGAC
Klk10	CAGTGCGAGCGTGACTATCAT	CAGTGGCTTATTTCTCCAGCAAT
Angptl4	GCATCCTGGGACGAGATGAAC	CCCTGACAAGCGTTACCACA
Bhlhe40	ACAAGCTGGTGATTTGTCGGG	AGTGACGAGCTGGGAAGATTT
Asb2	CCATTGACCAACGCACACTG	TGGATTTGTTAGAGATGTCAGGC
Hmox1	AAGCCGAGAATGCTGAGTTCA	GCCGTGTAGATATGGTACAAGGA
Bhlhe41	TGTGTAAACCCAAAAGGAGCTT	TGTTCGGGCAGTAAATCTTTCAG
Slc30a1	TCCTGATCCCTGCAAATCGTC	GTTGGGTCTAGGTAGAGCACC
Angptl6	TTGGGCGTCCAGAAGGAGAA	CAGTCCTCTAGGAGTATCAGCAG
Cd38	TCTCTAGGAAAGCCCAGATCG	AGAAAAGTGCTTCGTGGTAGG
Anxa8	TACCCGCCATACAGCTACGA	TGGTCCGAGAAGCTAGGATCT
Hyal1	ACCGGCCATTCATCACTGTTT	CTTGTTGGCAACCACATCGAA
Cyp24a1	CTGCCCCATTGACAAAAGGC	CTCACCGTCGGTCATCAGC
Mmp1b	GCTCATGCTTTTCTGCCAGG	TAGAATGGGAGAGTCCAAGGG

Table S1. Primer sequences used for qPCR
